# Pulmonary Hypoplasia Caused by Fetal Ascites in Congenital Cytomegalovirus Infection Despite Fetal Therapy

**DOI:** 10.3389/fped.2017.00241

**Published:** 2017-11-06

**Authors:** Kazumichi Fujioka, Ichiro Morioka, Kosuke Nishida, Mayumi Morizane, Kenji Tanimura, Masashi Deguchi, Kazumoto Iijima, Hideto Yamada

**Affiliations:** ^1^Department of Pediatrics, Kobe University Graduate School of Medicine, Kobe, Japan; ^2^Department of Obstetrics and Gynecology, Kobe University Graduate School of Medicine, Kobe, Japan

**Keywords:** congenital cytomegalovirus infection, pulmonary hypoplasia, fetal ascites, fetal therapy, newborn

## Abstract

We report two cases of pulmonary hypoplasia due to fetal ascites in symptomatic congenital cytomegalovirus (CMV) infections despite fetal therapy. The patients died soon after birth. The pathogenesis of pulmonary hypoplasia in our cases might be thoracic compression due to massive fetal ascites as a result of liver insufficiency. Despite aggressive fetal treatment, including multiple immunoglobulin administration, which was supposed to diminish the pathogenic effects of CMV either by neutralization or immunomodulatory effects, the fetal ascites was uncontrollable. To prevent development of pulmonary hypoplasia in symptomatic congenital CMV infections, further fetal intervention to reduce ascites should be considered.

## Introduction

Pulmonary hypoplasia is a rare and devastating morbidity among newborns, resulting in mortality up to 70% (1, 2). A pathological feature is an arrest in bronchial growth during fetal growth (3). The pathogenesis of pulmonary hypoplasia is generally categorized into three mechanisms, including thoracic compression, lack of fetal breathing movement, and loss of lung fluid (4). Many causal factors might lead to pulmonary hypoplasia, including intrathoracic masses, oligohydramnios, skeletal malformations, neuromuscular malformations, pleural effusion, cardiac lesions, abdominal wall defects, and chromosomal aberrations (2). Congenital cytomegalovirus (CMV) infection occurs in 0.2–2.0% of live-born infants (5), and is a major non-genetic cause of deafness and childhood neurodevelopmental disabilities (6). Therefore, aiming at improvement of fetal/infantile prognosis, Yamada and collaborators organized Japanese Congenital Cytomegalovirus Infection Immunoglobulin Fetal Therapy Study Group, and commenced multicenterded fetal therapy trial of hype-immunoglobulin injection into the peritoneal cavity for symptomatic congenital CMV infections since 2005. And, we have reported a promising results that 41.7% of symptomatic congenital CMV infections infants whose mothers received fetal therapies had no or minimal sequelae (7). Thus, recently, we have actively performed fetal therapy to prenatally diagnosed symptomatic congenital CMV infections with parental informed consent under approval of the institutional ethics boards of the Kobe University Hospital.

Symptomatic cases manifest various features at birth, including small-for-gestational age, microcephaly, thrombocytopenia, liver dysfunction, retinopathy, abnormal brain images, and abnormal auditory brainstem responses (6–10). However, lung complications are uncommon in congenital CMV infections (11).

We describe two cases of congenital CMV infections in patients who suffered from pulmonary hypoplasia due to massive fetal ascites despite fetal therapy and died soon after birth.

## Case History

### Case 1

**Figure 1:**
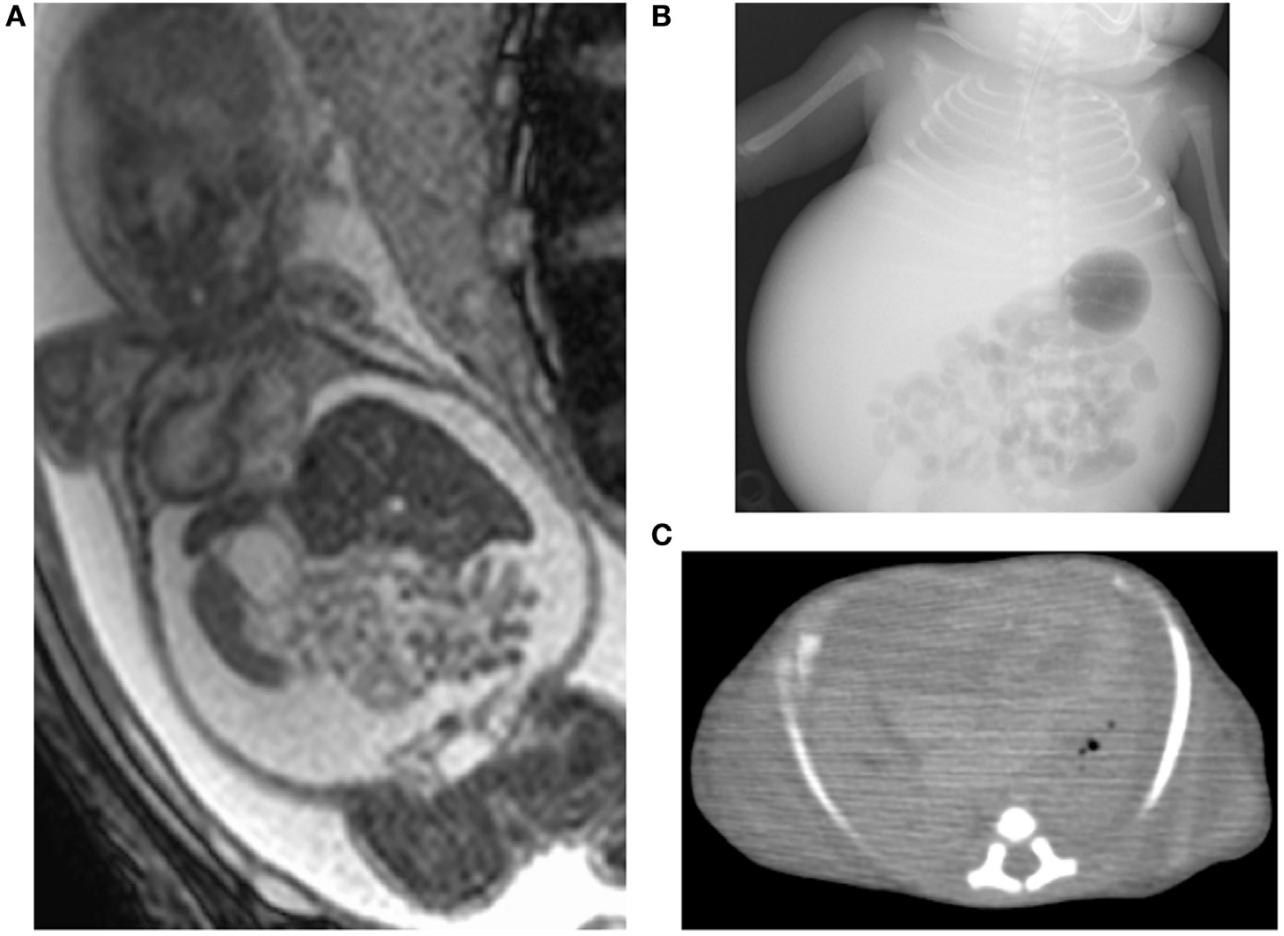
Images of case 1. Fetal magnetic resonance imaging shows massive ascites and compressed lungs **(A)**. Chest X-ray **(B)** and postmortem chest CT **(C)** show pulmonary hypoplasia.

A 27-year-old woman (gravida 2, para 1) underwent a routine ultrasound examination at 19 weeks’ gestation and fetal ascites was observed. She was referred to a tertiary center and her serology was positive for CMV-IgM. She was then transferred to our center for further treatment. She was confirmed as having primary CMV infection during pregnancy by positive CMV-DNA (3.4 × 10^5^ copies/ml) of fetal ascites and low CMV-IgG avidity (16.6%) at 20 weeks’ gestation. Fetal magnetic resonance imaging (MRI) at 27 weeks showed massive ascites and compressed low-intensity lungs (Figure 1A). We performed fetal therapies, including intravenous immunoglobulin to the mother [22, 24, and 25 gestational weeks (GW)], ascites removal followed by fetal intraperitoneal injection of immunoglobulin (20, 21, 23, 26, 27, and 29 GW) and albumin (22, 26, 28, 30, and 31 GW); and amniotic fluid removal for polyhydramnios, which occurred since 28 weeks (28, 30, and 31 GW). At 31 GW and 0 days, she gave birth to a female neonate (birth weight of 1,824 g; Apgar scores of 4 and 6 at 1 and 5 min, respectively) *via* emergent cesarean section because of signs of threatened premature delivery after cordocentesis. The neonate was tracheally intubated soon after birth, and her heart rate gradually increased. Her abdomen was massively distended with palpable fluctuation, but no petechiae were detected. A blood test showed mild leukopenia (3,800/μl), anemia (86 g/l), thrombocytopenia (6.1 × 10^4^/μl), hypogammaglobulinemia (3.46 g/l), and hypoalbuminemia (24 g/l). Despite the normal aspartate aminotransferase (66 IU/l) and alanine aminotransferase (5 IU/l) levels, the level of total protein (29 g/l), fibrinogen (680 mg/l), and prothrombin time (<10%) were significantly decreased, suggesting liver failure from congenital CMV infections. An X-ray showed marked reduction in lung volume (Figure 1B), with an increase in abdominal volume by massive ascites. Abdominal echo revealed hepatomegaly and brain echo revealed periventricular hyperechogenicity but could not detect ventriculomegaly and calcifications.

We removed 300 ml of peritoneal fluid by peritoneocentesis to lower the lung compression. However, her respiratory status gradually deteriorated, despite the highest ventilator settings, with signs of persistent pulmonary hypertension. Therefore, we started inhaled nitric oxide 6 h after birth, but she developed pneumopericardium at 8 h. At 14 h after birth, she eventually died because of pulmonary hypoplasia. Autopsy imaging showed ventriculomegaly and intracranial calcification, in addition to a reduced lung volume (Figure 1C). Positive CMV-DNA (blood: 66 copies/10^6^ white blood cells, urine: 1.2 × 10^7^ copies/ml) confirmed congenital CMV infection.

### Case 2

**Figure 2:**
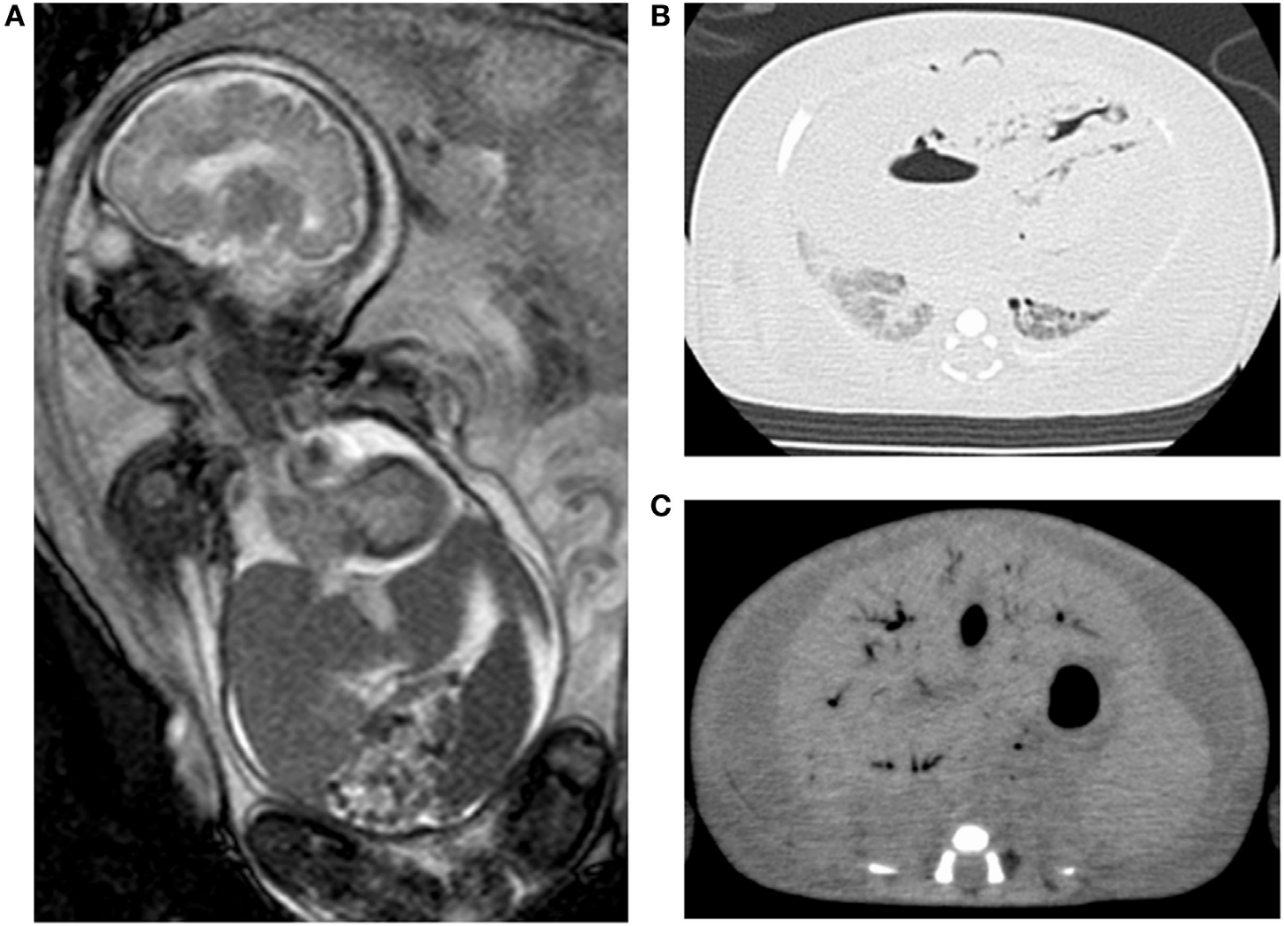
Images of case 2. Fetal magnetic resonance imaging shows massive ascites, hepatomegaly, and compressed lungs **(A)**. Postmortem chest **(B)** and abdominal CT **(C)** show pulmonary hypoplasia and massive ascites with hepatomegaly.

A 30-year-old woman (gravida 3, para 1) had fetal ascites detected at 22 GW by occasional ultrasound with the complaint of abdominal distension. Further investigation showed fetal pericardial effusion and positive CMV-IgM. Because of positive CMV-DNA in the amniotic fluid (3.4 × 10^6^ copies/ml), she was transferred to our center for further treatment at 24 GW and 4 days. Fetal MRI at 30 weeks showed massive ascites, hepatomegaly, pericardial effusion, and compressed low-intensity lungs (Figure 2A). We performed fetal therapies, including intravenous immunoglobulin to the mother (24, 25, 26, and 30 GW), ascites removal followed by fetal intraperitoneal injection of immunoglobulin at 28 GW and albumin plus packed red blood cells at 29 GW to treat fetal anemia. At 31 GW and 2 days, she gave birth to a female neonate (birth weight, 2,236 g; Apgar scores of 1 and 1 at 1 and 5 min, respectively) *via* emergent cesarean section because of non-reassuring fetal status. The neonate was heavily edematous and had bradycardia at birth. Her condition was diagnosed as hydrops fetalis based on the generalized edema and massive ascites. She was resuscitated with manual ventilation under inhaled nitric oxide and chest compression. However, she did not respond to resuscitation and died 20 min after birth. A blood test showed leukocytosis (31,300/μl), anemia (41 g/l), thrombocytopenia (2.4 × 10^4^/μl), hypogammaglobulinemia (1.51 g/l), hypoalbuminemia (6.0 g/l), and increased aspartate aminotransferase levels (650 IU/l). Abdominal echo revealed hepatomegaly with hyperechoic lesions suggesting hemorrhage in the liver, and brain echo revealed mild ventriculomegaly and hyperechoic lesions suggesting calcifications. Autopsy imaging showed pulmonary hypoplasia, hepatomegaly, ascites, and intracranial calcification (Figures 2B,C). Positive CMV-DNA (blood: 1.6 × 10^3^ copies/10^6^ white blood cells, tracheal aspirates: 4.5 × 10^4^ copies/ml) confirmed congenital CMV infection.

## Discussion

Pulmonary hypoplasia is a rare condition, affecting 9–11/10,000 live births (1). The pathophysiology of pulmonary hypoplasia is a reduction in the number of lung cells, airways, and alveoli, resulting in a decrease in organ size and weight. These factors are highly correlated with insufficient gas exchange (4). Diagnosis of pulmonary hypoplasia is based on clinical findings of respiratory distress occurring almost secondary to other fetal anomalies (1, 10). In addition, low-intensity fetal lung on MRI could be the clues of pulmonary hypoplasia (12). There have been no sufficient data regarding its impact on the neurodevelopmental outcome; however, favorable outcomes at 5 years of ages were reported in congenital diaphragmatic hernia, which is often associated with pulmonary hypoplasia (13). The most feasible pathogenesis of pulmonary hypoplasia in our cases is thoracic compression *via* massive ascites, which occurred from early pregnancy. The greatest impairment of lung development occurs when compression occurs in the lungs during the last trimester (4). The fetal lungs were highly compressed at this time in our cases. Extrinsic thoracic compression usually occurs in oligohydramnios, where the maternal uterine wall directly compresses the fetal thorax (4). Our case 2 suffered from hydrops fetalis, and Page and Stocker reported this as the cause of pulmonary hypoplasia (14); however, their cases were complicated with large pleural effusions or renal dysplasia; two main causes of pulmonary hypoplasia *via* thoracic compression or loss of lung fluid. Our case 2 showed only mild pericardial effusions but not pleural effusions, and we considered that those could not affect the lung development significantly as space occupying lesions of the chest. Thus, we believe that not hydrops fetalis itself, but its underlying mechanisms to develop pulmonary hypoplasia is more important. In our cases, the volume of ascites was so large that the fetal abdominal wall might not have been able to extend further, resulting in strong compression of the fetal thorax. Although fetal ascites is an uncommon complication of congenital CMV infections, overt systemic disease might occur as hepatosplenomegaly and ascites in the fetus as a result of liver insufficiency (15).

In lung complications of congenital CMV infections, a few patients suffering from persistent pulmonary hypertension have been reported (16–18). However, their etiology was hypothesized to be interstitial pneumonia or vasculitis, and not pulmonary hypoplasia. Similar to our cases, Stocker reported an autopsy case of congenital CMV infection presenting as massive ascites (28% of total body weight) with secondary pulmonary hypoplasia (26% of expected weight) that was diagnosed at postmortem (19). In his study, CMV inclusion bodies were detected within the nucleus and cytoplasm of the epithelial cells of the bile duct. Although we could not perform the autopsy of the lungs due to unavailability of parental consents, fetal MRI and autopsy imaging could add sufficient information regarding pulmonary hypoplasia.

Despite aggressive neonatal resuscitation, we could not rescue our patients. Additionally, multiple fetal therapies failed to prevent development of pulmonary hypoplasia. Fetal treatment with hyper-immunoglobulin was supposed to have neutralizing effects by inhibiting the replication of CMV, or immunomodulatory effects by decreasing the immune cells associated with the production of inflammatory cytokines which can contribute to immune-mediated fetal damages (20–22). We have previously reported that (1) immunoglobulin fetal therapy could decrease CMV DNA copy numbers in fetal ascites and (2) CMV viral load is weaker in fetal ascites than in amniotic fluid in congenital CMV infections (7). With regard to viral load, CMV-DNA copy numbers in amniotic fluid of our two cases are comparable with those in previous reports where fetal therapy caused a good outcome (7). Therefore, we believe that fetal treatment modality, which consisted of ascites removal followed by administration of immunoglobulin or albumin, was effective in controlling viral load, but not effective for controlling the volume of fetal ascites.

## Concluding Remarks

Pulmonary hypoplasia due to fetal ascites is a rare, but an important, cause of neonatal death in congenital CMV infections. Further fetal intervention to control fetal ascites should be considered.

## Ethics Statement

Parental written informed consent was given to present cases. The fetal therapy protocol was approved by the institutional ethics boards of the Kobe University Hospital.

## Author Contributions

KF, IM, and KN managed the patients, contributed to the conception of the study, and drafted the manuscripts; MM, KT, and MD managed the mothers and performed the fetal therapy; and KI and HY critically reviewed the manuscript. All authors read and approved the final manuscript.

## Conflict of Interest Statement

The authors declare that the research was conducted in the absence of any commercial or financial relationships that could be construed as a potential conflict of interest. The reviewer MV and handling editor declared their shared affiliation.
